# Validation of the Arabic version of the brief irritability test (Ar-BITe) in non-clinical adolescents

**DOI:** 10.1186/s12888-023-05211-y

**Published:** 2023-09-26

**Authors:** Feten Fekih-Romdhane, Vanessa Azzi, Rabih Hallit, Diana Malaeb, Mariam Dabbous, Fouad Sakr, Sahar Obeid, Souheil Hallit

**Affiliations:** 1 The Tunisian Center of Early Intervention in Psychosis, Department of psychiatry “Ibn Omrane”, Razi hospital, Manouba, 2010 Tunisia; 2 Faculty of Medicine of Tunis, Tunis El Manar University, Tunis, Tunisia; 3 School of Medicine and Medical Sciences, Holy Spirit University of Kaslik, P.O. Box 446, Jounieh, Lebanon; 4 Department of Infectious Disease, Bellevue Medical Center, Mansourieh, Lebanon; 5 Department of Infectious Disease, Notre Dame des Secours, University Hospital Center, Byblos, Lebanon; 6 College of Pharmacy, Gulf Medical University, Ajman, United Arab Emirates; 7 School of Pharmacy, Lebanese International University, Beirut, Lebanon; 8 École Doctorale Sciences de la Vie et de la Santé, Université Paris-Est Créteil, Institut Mondor de Recherche Biomédicale, Créteil, France; 9 Social and Education Sciences Department, School of Arts and Sciences, Lebanese American University, Jbeil, Lebanon; 10 Psychology Department, College of Humanities, Effat University, Jeddah, 21478 Saudi Arabia; 11 Applied Science Research Center, Applied Science Private University, Amman, Jordan; 12 Research Department, Psychiatric Hospital of the Cross, Jal Eddib, Lebanon

**Keywords:** Irritability, BITe, Arabic, Psychometric properties, Adolescents

## Abstract

**Background:**

Despite the substantial clinical relevance of irritability in the development and maintenance of several mental disorders and its negative effects on functioning, no valid and reliable measures are available yet to identify the presence and consequences of irritability as a distinct construct among the Arabic-speaking populations. To bridge this gap, and help advance this field in the under-researched Arab region, we aimed to validate an Arabic-language version of the Brief Irritability Test (BITe).

**Methods:**

Eligible participants were native Arabic-speaking non-clinical adolescents from Lebanon; 527 participants aged 15.73 ± 1.81 years (56% females) completed the survey.

**Results:**

Utilizing the Confirmatory Factor Analysis approach, we found that the five items of the Arabic BITe loaded into a single factor structure. The scale showed excellent reliability, as both Cronbach’s alpha and McDonald’s omega coefficient values were of 0.88. Multi-group analyses showed invariance across sex groups in our sample at the configural, metric, and scalar levels. Female adolescents exhibited higher BITe scores than their male counterparts (14.01 vs. 13.25), but this difference did not reach the statistical significance. Good concurrent validity was supported based on positive correlations between irritability scores and measures of aggression, anger and hostility (r Pearson’s coefficients ranging from 0.35 to 0.42), as well as positive correlations with insomnia symptoms scores.

**Conclusion:**

The present findings allow us to conclude that the Arabic version of the BITe is a unidimensional, reliable, valid, brief, and economic self-report measure of the irritability construct for both male and female Arabic-speakers. Providing an Arabic validated version of the BITe will hopefully foster the research efforts of the Arab scientific community in this area, and promote the implementation of timely, evidence-informed and culturally-sensitive mental health interventions that appropriately address irritability-related problems and consequences among Arab young populations.

## Background

Irritability refers to an emotion characterized by a liability to experience increased sensitivity to negative emotional stimuli, which may lead to affective (i.e. annoyance, anger, frustration upon little provocation) and/or behavioral (i.e. aggression) responses [1–3]. Although irritability may be viewed as normative, it could be potentially related to psychopathology [4]. The Diagnostic and Statistical Manual of Mental Disorders, Fifth Edition (DSM-5) clearly states that irritability is a shared feature within numerous psychiatric disorders, and that when severe and persistent, it represents the central feature of Disruptive Mood Dysregulation Disorder [5]. In addition, extensive amount of research has shown that heightened irritability exists across a variety of behavioral and psychological problems, including attention deficit/hyperactivity disorder [6], unipolar depression [7], bipolar disorder [8], anxiety disorders (e.g., [9]), suicide [10], addiction [11], sleep problems [12], personality disorders [13, 14], conduct disorder [15], and oppositional defiant disorder [16]. Irritability was also consistently found to have long-lasting effects on functioning in various domains (i.e. educational, financial and social) (e.g., [17]). Furthermore, irritability has proven to have a developmental significance in children and adolescents, as it predates the emergence of externalizing and internalizing disorders in these populations [18, 19], and prospectively predicts severity and chronicity of impairment in those who are affected by any psychopathology [2, 7, 20–23]. All these findings broadened evidence on the transdiagnostic nature of irritability [15]. This implies that the development of psychological interventions targeting the remediation of irritability as a transdiagnostic factor may positively affect all the disorders it is related to [24]. Taken together, these data underline the major importance of screening and monitoring irritability validly and reliably in both clinical and non-clinical populations in order to promote its identification, prevention, and treatment.

There has been a drastic increase in research interest in irritability over the past years, which was mirrored by the development and use of several self-report measures to capture the construct (e.g., Caprara Irritability Scale [25], the Born-Steiner Irritability Scale (BSIS) [26], the Irritability Questionnaire (IRQ) [27], the Affective reactivity index Self-Report [28]). However, many of these measures were criticized for several reasons. One of the reasons relates to the definition of the concept of irritability. Some tools (e.g., [25, 29]) conceptualize irritability as a stable trait that shows temporal stability [21, 30, 31] and heritability [31, 32]. Other tools, however, consider irritability as a mood state (e.g., [26–28, 33, 34]), and therefore as temporary (i.e. lasting for hours, days, or weeks), fluctuating symptom [5], occurring in the absence of “justifiable” triggers and “clear antecedents” ( [3], p. 96). Overall, it is clear that both genetic and environmental influences play a role for irritability, and therefore that irritability has both trait and state properties [35]. Another reason is that the vast majority of irritability research has relied on measures that are not designed for assessing irritability as a distinct construct [1], but rather as a subscale within larger measures (e.g., the Aberrant Behavior Checklist [36]). Furthermore, most of these measures’ items reflect other constructs, such as hostility, aggression, and anger, rather than the construct of irritability [3], which may hinder the reliable and valid identification of irritability and its related psychopathology, as well as the possibility of finding adequate interventions [1–3]. Because of these shortages, many researchers underscored the need for designing measurement instruments to assess irritability in a more precise and comprehensive way [1–3, 21].

To address the above-mentioned gaps, Holtzman et al. [37] developed a short self-report measure that conceptualizes irritability as a state, i.e. the Brief Irritability Test (BITe). The BITe assesses irritability within the last two weeks through five items, which are displayed in Table 1. The construct of irritability as defined by the BITe refers to a range of thoughts, feelings, behaviors, and sensations that are clearly distinct from other related constructs, including depression, anger, and hostility. The developers of the scale started by generating an initial pool of 63 candidate items, of whom thirty-five were obtained from two existing self-report irritability measures (i.e., the 21-item Irritability Questionnaire [27] and the 14-item Born–Steiner Irritability Scale [26]). The rest of items were developed based on a content analysis of published definitions of irritability, a review of previous irritability measures, and qualitative interviews with community adults [37]. The scale was then validated in a sample of undergraduate students and outpatients with chronic pain, using item response theory. The BITe demonstrated excellent psychometric properties in non-clinical (i.e., university students) and clinical (i.e., chronic pain patients) Canadian samples in terms of internal consistency (α = 0.88) and concurrent validity as attested through positive correlations with other measures of irritability (i.e. IRQ, BSIS), anger, aggression, and hostility [37, 38]. In addition, Holtzman et al. [37] reported a significant correlation between measures of irritability and pain interferences of life, which reflects physiological capabilities such as sleep [39]. These findings are in agreement with available evidence that irritability is related to physiological states, including insomnia [40]. One potential advantage to utilizing BITe is its briefness, which makes it feasible to be implemented in clinical and research contexts where questionnaire length or administration time are limited. The BITe is preferred over other measures not only for being more economical and time-efficient, but also for having stronger content validity, which signifies that it is more specific in differentiating irritability from similar, related but distinct constructs (such as aggression and anger) and in identifying qualities linked to irritability [3, 37, 38]. Since its development, the BITe has been translated and validated in other-than-English languages, including French [41], Spanish [42], and Turkish [43]. However, as far as we are aware of, there is no Arabic version of the BITe available.

### The present study

In this study, we sought to contribute to the scientific literature on irritability, by offering a validated Arabic version of the BITe for use among Arabic-speaking populations. This goal was motivated by several considerations. First, despite the increasing attention paid to comprehending the mechanisms underlying the causes and effects of irritability in different parts of the world, we could find no studies addressing this question in an Arabic-speaking population from the Arab region. The scarcity of high-quality data on irritability emerging from Arab countries may hamper the scientific progress in this field. Second, there has been some limited and preliminary evidence on the cross-cultural differences in reporting irritability, and calls for future research on cross-national and cross-cultural conceptualizations of irritability [44]. However, psychometrically sound, adapted, and culturally-tailored measurement instruments need to be available before research can move forward. Third, Arab countries have the most youthful age structure in the world, with around 60% of the population being under 25 years old [45]. This creates many challenges to taking care of the mental health needs of Arab youth, especially in the context of the ongoing unstable political, economic, and social climate in the Arab region that has led to higher-than-expected mental health-related burden [46]. Epidemiological research has revealed that the peak age of emergence of mental health disorders was 14.5 years, with 34.6% and 48.4% of individuals having onset of any mental disorders before the ages of 14 and 18, respectively [47]. Given the transdiagnostic nature of irritability and its developmental significance in youth, there appears to be a strong need for providing psychometrically sound measures of this construct for this vulnerable group in Arab countries.

To this end, we aimed through the current study to examine the psychometric properties of an Arabic translation of the BITe in a sample of non-clinical Arabic-speaking adolescents from Lebanon. We hypothesized that the Arabic BITe will (1) yield a unidimensional factor structure, (2) be invariant across sex (male vs. female), and (3) demonstrate good composite reliability as evidenced through Cronbach’s alpha and McDonald’s omega coefficient values greater than 0.70. We also expected that the Arabic version of the BITe will show good concurrent validity results with measures of aggression, anger, and hostility, and insomnia.

## Methods

### Participants and procedures

A total of 527 adolescents completed the survey (mean age: 15.73 ± 1.81; 56% females). A convenient sampling method (snowball technique) was used to collect data during April-May 2023. After completing a training with the research team, five university students were asked to collect data via a Google Form link; they were asked to forward the link to people they know, who in turn were asked to forward the link to other family members and friends. Inclusion criteria for participation included being of a resident and citizen of Lebanon and aged between 12 and 18 years. Excluded were those who refused to fill out the questionnaire. Internet protocol (IP) addresses were examined to ensure that no participant took the survey more than once. Participants were asked in the introductory paragraph to take their parents’ consent before filling the survey. After providing digital informed consent, participants were asked to complete the instruments described above, which were presented in a pre-randomized order to control for order effects. The survey was anonymous and participants completed the survey voluntarily and without remuneration [63].

### Translation Procedure

The Brief Irritability Test Scale was translated using the forward-backward method. A Lebanese translator who had no connection to the study translated the English text from English into Arabic. The Arabic version was then translated back into English by a Lebanese psychologist who is fully functional in English. Any literal or specific translation was balanced by the translation team. A committee of experts made up of the study team, one psychologist, one psychiatrist, and the two translators examined the original and translated English versions to find/eliminate any discrepancies and ensure the translation’s accuracy. [48]. In order to establish conceptual equivalence between the original and Arabic scales in both contexts, a measure adaptation for the Arab context was carried out. This adaptation intended to identify any potential misinterpretation of the items’ wording as well as the ease of items interpretation [49]. Conceptual equivalence was tested with 20 adolescent students with bilingual skills. The original instrument was carefully assessed with respect to its conceptual equivalence to the Arab culture, allowing to evaluate whether the five items corresponded to the values and beliefs of Arab adolescents. Therefore, amendments in the Arabic BITe were made based on the results of the first round of conceptual equivalence and undertaking the second round of assessment with different participants. After the scale was translated and adapted, pilot research with 30 participants was conducted to make sure all of the questions were well understood; no changes were made as a result of the pilot study.

### Measures

#### Demographics

Participants were asked to provide their demographic details consisting of age and sex.

#### Brief Irritability Test

The BITe is a five-item self-report tool that assesses irritability over the last two weeks [37]. Each item is rated on a 6-point Likert scale ranging from 1 (never) to 6 (always). A total score is calculated by summing the score of each item, with higher scores reflecting more irritability. depicting the level of irritability of a subject.

#### The Insomnia Severity Index (ISI)

This scale measures insomnia symptoms severity through seven items [50]. We used the validated Arabic version of the ISI [51]. The scale is rated on a four-point Likert scale. Higher scores indicate more severe insomnia The present sample yielded internal consistency coefficients of ω = 0.68 and α = 0.59, compared to α = 0.83 in the validation study). We chose this scale to evaluate the validity of the BITe scale since poor sleep is known to be associated with irritability [52].

#### The Buss–Perry Aggression Questionnaire-Short Form (BPAQ-SF)

Validated in Arabic [53], the BPAQ-SF [54] is a short version of the BPAQ, and it contains 12 items rated on a 5-point Likert scale. Higher scores indicate higher levels of aggression. The items yield four subscales assessing physical aggression (ω = 0.78 and α = 0.78), verbal aggression (ω = 0.66 and α = 0.63), anger (ω = 0.77 and α = 0.76), and hostility (ω = 0.78 and α = 0.78) in this study. In the validation study, the Cronbach’s alpha values were as follows: physical aggression (α = 0.66), verbal aggression (α = 0.55), hostility (α = 0.72), and anger (α = 0.71).

### Analytic strategy

#### Confirmatory factor analysis

There were no missing responses in the dataset. Since the structure of the scale is known, we used data from the total sample to conduct a CFA using the SPSS AMOS v.29 software. Our intention was to test the unidimensional model obtained from the original study [37]. The normed model chi-square (χ²/df), the Steiger-Lind root mean square error of approximation (RMSEA), the Tucker-Lewis Index (TLI) and the comparative fit index (CFI). Values ≤ 5 for χ²/df, and ≤ 0.08 for RMSEA, and 0.90 for CFI and TLI indicate good fit of the model to the data [55]. The absence of multicollinearity was verified through tolerance values > 0.2 and variance inflation factor (VIF) values < 5. Multivariate normality was not verified at first; therefore, we performed non-parametric bootstrapping procedure (available in AMOS). Evidence of convergent validity was assessed in this subsample using the average variance extracted (AVE) values of ≥ 0.50 considered adequate (Malhotra & Dash, 2011).

#### Measurement invariance across sex

To examine sex invariance of BITe scores, we conducted multi-group CFA [56] using the total sample. Measurement invariance was assessed at the configural, metric, and scalar levels. Configural invariance implies that the latent BITe variable(s) and the pattern of loadings of the latent variable(s) on indicators are similar across gender (i.e., the unconstrained latent model should fit the data well in both groups). Metric invariance implies that the magnitude of the loadings is similar across gender; this is tested by comparing two nested models consisting of a baseline model and an invariance model. Lastly, scalar invariance implies that both the item loadings and item intercepts are similar across gender and is examined using the same nested-model comparison strategy as with metric invariance [56, 57]. Measurement invariance was determined if Δχ² was not significant (*p* > .05), ΔCFI ≤ 0.010 and ΔRMSEA ≤ 0.015 or ΔSRMR ≤ 0.010 [56]. If invariant, we aimed to check for a difference in NS scores in terms of sex using the Student *t*-test.

### Reliability and validity testing

Composite reliability in both subsamples was assessed using McDonald’s ω and Cronbach’s alpha [58], with values greater than 0.70 reflecting adequate composite reliability. The total BITe scores followed a normal distribution, with skewness and kurtosis values varying between − 1 and + 1 [59]. To assess concurrent validity, we examined bivariate correlations between NS scores and the other scales included in the survey using the Pearson test. Based on Cohen [60], values ≤ 0.10 were considered weak, ~ 0.30 were considered moderate, and ~ 0.50 were considered strong correlations.

## Results

### Confirmatory factor analysis

A CFA was conducted on the total sample; the fit indices were acceptable as follows: χ^2^/df = 10.76/5 = 2.15, *p* < .001, RMSEA = 0.047 (90% CI 0.001, 0.086), SRMR = 0.012, CFI = 0.996, and TLI = 0.992. The standardised estimates of factor loadings were all adequate (see Table 1). The convergent validity for this model was adequate, as AVE = 0.77 (ω = 0.88 and α = 0.88).

**Table 1:** Items of the Brief Irritability Test in English and Standardised Estimates of Factor Loadings from the Confirmatory Factor Analysis (CFA) in the total sample

Item	CFA
(1) I have been grumpy	0.77
(2) I have been feeling like I might snap	0.54
(3) Other people have been getting on my nerves	0.86
(4) Things have been bothering me more than they normally do	0.81
(5) I have been feeling irritable	0.85

### Measurement invariance across sex

Indices in Table 2 indicate that configural, metric, and scalar invariance was supported across sex in the total sample. No significant difference was found between females (*M* = 14.01, *SD* = 5.12) in terms of BITe scores compared to males (*M* = 13.25, *SD* = 4.29), *t*(525) = -1.853, *p* = .064.

**Table 2:** Measurement Invariance Across Sex

Model	χ²	*df*	χ²/ *df*	CFI	RMSEA	SRMR	Model Comparison	Δχ²	ΔCFI	ΔRMSEA	ΔSRMR	Δ*df*	*p*
Configural	20.70	10	2.07	0.992	0.045	0.028							
Metric	25.40	14	1.81	0.992	0.039	0.031	Configural vs. metric	4.7	< 0.001	0.006	0.003	4	0.319
Scalar	26.35	18	1.46	0.994	0.030	0.031	Metric vs. scalar	0.95	0.002	0.009	< 0.001	4	0.917

Note. CFI = Comparative fit index; RMSEA = Steiger-Lind root mean square error of approximation; SRMR = Standardised root mean square residual; χ²/df = normed model chi-square

### Concurrent validity

Higher BITe scores were significantly and positively correlated with more insomnia severity (*r* = .23, *p* < .001), physical aggression (*r* = .39, *p* < .001), verbal aggression (*r* = .35, *p* < .001), anger (*r* = .42, *p* < .001) and hostility (*r* = .36, *p* < .001).

## Discussion

Despite the substantial clinical relevance of irritability in the development and maintenance of several mental disorders and its negative effects on functioning [1, 61], no valid and reliable measures are available yet to identify the presence and consequences of irritability as a distinct construct among the Arabic-speaking populations. To bridge this gap, and help advance this field in the under-researched Arab region, we aimed to validate the Arabic version of the BITe, which was suggested to be the “more precise and reliable tool for measuring irritability”, and thus the most beneficial for use in youth populations [38]. As expected, findings revealed that the Arabic BITe has a good internal consistency, a unidimensional factor structure, and was invariant across sex groups. In addition, we found that the scale demonstrated good concurrent validity. Overall, the Arabic BITe is a brief, low-cost, valid, and reliable measure. We thus endorse its use as a measurement instrument for assessing irritability among Arabic-speaking adolescents.

As for factorial validity, and utilizing the CFA approach, we found that the five items of the Arabic BITe loaded into a single factor, thus supporting the originally proposed factor model of the scale [37]. The other linguistic versions available of the BITe were also able to replicate the unidimensional factor solution of the BITe among French-speaking Belgian [41] and Spanish-speaking Paraguayan [42] non-clinical adults, as well as Turkish-speaking undergraduate and postgraduate students [43]. The one-factor structure of the BITe was also supported in other samples and countries (e.g., US community adults [62]). Mean total scores found in the present sample were of 13.68. Mean scores reported in previous studies were of 12.95 in Canadian [37], 12.99 in Turkish [43], 15.36 in French [41], and 16.51 in Spanish [42] populations. These differences in BITe scores may be explained by differences in the samples’ characteristics, by cultural differences specific to each country [44], and/or by distinct functioning of the BITe across groups and countries [63, 64]. Therefore, cross-cultural and cross-national comparisons cannot be meaningful and conclusive unless measurement invariance of the BITe across countries is achieved.

The Arabic BITe showed excellent reliability, as both Cronbach’s alpha and McDonald’s omega coefficient values were of 0.88. This is consistent with the original English (α = 0.91) and other language (i.e. Turkish α = 0.86, [43]; French α = 0.80, [41]; Spanish α = 0.91 [42]) versions. Multi-group analyses indicated invariance across sex groups in our sample at the configural, metric, and scalar levels. This finding was expected, as the BITe was originally specifically designed to be suitable for both males and females [37]. As such, the developers of the scale demonstrated that there is no gender bias on each of the five BITe items [37]. These findings suggest that male and female respondents understand and interpret items in a similar way, and that the Arabic BITe operates similarly across gender groups. This implies that the Arabic BITe allows for robust investigation of gender differences in irritability among Arabic-speaking people. In our sample, no significant differences were found between male and female adolescents in BITe scores. This is in contrast with previous literature that consistently reported higher rates of irritability in females compared to males [42, 44, 65], which may be explained by cultural factors.

Good concurrent validity was supported for the Arabic BITe based on positive correlations between irritability scores and measures of aggression, anger and hostility (r Pearson’s coefficients ranging from 0.35 to 0.42). Similar positive correlations between these constructs were also observed in other validation studies of the BITe (e.g., [41–43]).These findings provide additional empirical support for the high specificity of the BITe in measuring the irritability construct [37]. Furthermore, good concurrent validity of the Arabic BITe was evidenced by positive correlations with insomnia symptoms scores. This is in agreement with previous evidence suggesting irritability as a transdiagnostic factor that can be observed across different mental problems, including sleep disturbances [12, 66]. In sum, these findings provide further evidence for the clinical relevance of assessing the irritability construct particularly in adolescents.

### Limitations and research perspectives

This study is not without limitations. First, we adopted a convenience snowball sampling technique, which may have affected the representativeness of our sample. In addition, our participants were recruited online. While this method allowed access to a relatively large sample of adolescents and ensured their anonymity, it might limit the generalizability of our results to adolescents who do not have access to technology devices and the Internet. The generalizability of our conclusions may also hindered by the inclusion of an exclusively adolescent and non-clinical sample from one Arab country. Future studies should consider testing the psychometric properties of the Arabic BITe in different age groups to allow for assessing the irritability construct across the life span. In addition, the robustness of the Arabic BITe still needs to be verified in clinical populations and other Arab countries. The internal consistency of the insomnia severity index and verbal aggression subscale was not optimal; results should be interpreted with caution. A main concern encountered in all studies where data is gathered via an online link, s the accuracy of information as well as consent reliability. Finally, other important psychometric characteristics of the BITe were not explored in the present study (e.g., Test-retest reliability) and should be subject of future investigations.

## Conclusion

The present study showed preliminary evidence to support that the Arabic version of the BITe, as a unidimensional measured, could produce reliable and valid measures of irritability in adolescents. However, more work is warranted for drawing valid clinical and research recommendations on its use. Providing an Arabic validated version of the BITe will hopefully foster the research efforts of the Arab scientific community in this area, and promote the implementation of timely, evidence-informed and culturally-sensitive mental health interventions that appropriately address irritability-related problems and consequences among Arab young populations, at least in Lebanon.

## Data Availability

The datasets generated and/or analyzed during the current study are not publicly available due to the restrictions from the ethics committee but are available from the corresponding author on a reasonable request.
